# Electrophysiological and Behavioral Indices of the Role of Estrogens on Memory Processes for Emotional Faces in Healthy Young Women

**DOI:** 10.3389/fnbeh.2019.00234

**Published:** 2019-10-01

**Authors:** Antonella Gasbarri, Mario D’Amico, Benedetto Arnone, Carla Iorio, Francesca Pacitti, Sabatino Ciotti, Paola Iorio, Assunta Pompili

**Affiliations:** ^1^Department of Biotechnological and Applied Clinical Sciences, University of L’Aquila, L’Aquila, Italy; ^2^Fondazione Santa Lucia, IRCCS, Rome, Italy

**Keywords:** estrogens, memory, event-related potentials, emotional face expressions, menstrual cycle

## Abstract

It is well known that estrogens influence cognitive activities, such as memory, and emotional states. The objective of the present study was to investigate the role of estrogens in the short-term memory processing of basic emotional face expressions, by means of event-related potentials (ERPs) and a recognition memory (RM) behavioral task. Healthy young women were divided into a periovulatory (PO) group, characterized by high levels of estrogens and low levels of progesterone, and an early follicular (EF) group, characterized by low levels of both estrogens and progesterone. During the RM task, all subjects viewed images of faces expressing six basic emotions (happiness, anger, disgust, sadness, surprise, fear) and one neutral expression while their electrophysiological activity was recorded. We considered P300 components, amplitude, and latency in response to each stimulus. Soon after the presentation of each stimulus face, a target image was presented, consisting of two faces, one of which was the same, while the other was a chimerical face, obtained by mixing the upper or lower halves of the faces of the stimulus image with a different emotion. The subjects had to choose between the two alternatives, and the reaction time (RT) and accuracy of response (RM errors) were measured. The main findings of this study showed that P300 amplitudes are significantly higher in response to the expressions of happiness, but significantly lower for sadness, in PO compared to EF. The P300 data are consistent with performance in the RM task and with the measures of RT. The interest in the emotion of happiness, unlike sadness, during the PO phase may reflect the evolutionary significance of female sex hormones linked to mating behavior.

## Introduction

The influence of estrogens, the principal female sex hormones, on learning and memory is of particular interest due to increasing average life expectancies. It is well documented in animal (Pompili et al., 2010; Frick et al., 2018) and human studies in both physiological and pathological conditions (Pompili et al., 2012; Bean et al., 2014; Kwakowsky et al., 2016; Hampson, 2018).

The effect of estrogens on the brain is well known, with the widespread presence of receptors (ERs), ERα, ERβ, and G-protein coupled ER in regions crucial for learning, memory, and emotion, including the cerebral cortex, the hippocampal formation (HF), and amygdala (Osterlund et al., 2000a,b; Gasbarri et al., 2012; Lymer et al., 2018). Through their ERs, estrogens act rapidly on neurotransmitter systems (Paletta et al., 2018), such as acetylcholine (Norbury et al., 2007; Newhouse and Dumas, 2015), considered a basic neurotransmitter in the regulation of learning and memory, but also on serotonin (Amin et al., 2005), catecholamine (Leranth et al., 2000), and GABA (Mukherjee et al., 2017). Moreover, estrogens have a profound effect on dendritic and synaptic morphology (Protopopescu et al., 2008; Spencer et al., 2008; Kato et al., 2013; Frankfurt and Luine, 2015; Luine et al., 2018) and on long-term potentiation (Smejkalova and Woolley, 2010), mechanisms closely related with encoding, and the subsequent consolidation of new memories.

Memory processing of emotional stimuli consisting of faces is a basic need. In fact, one of the most important social skills is the ability to interpret the moods and feelings of other people, and facial emotional expressions provide numerous different socially important signals, and so likely represent the most important visual stimuli in the human environment, both from a biological and a social point of view (Ekman, 1999; Adolphs, 2003; Keltner et al., 2003; Adams et al., 2017).

Therefore, it is not surprising that the recognition of facial expressions is altered in many affective, neurological, and psychiatric disorders (Kohler et al., 2010; Argaud et al., 2018; Borgsted et al., 2018; Sfärlea et al., 2018).

Experimental evidences show sex differences in the processing of facial expressions from adolescence onwards (Lee et al., 2013). Numerous studies suggest that women have superior performance in facial expression processing and recognition (Lewin and Herlitz, 2002; Güntekin and Başar, 2007; Rehnman and Herlitz, 2007; McBain et al., 2009; Wang, 2013), a skill called “the female advantage” (Hampson et al., 2006). It has been hypothesized that women have a greater empathic ability and a greater interest in social characteristics, which allows them to pay more attention to facial stimuli (Proverbio, 2017).

It is reasonable that these differences could also be explained from a hormonal point of view, taking into account the fact that estrogens seem to play a role in social learning and memory (Choleris et al., 2012; Karlsson et al., 2016; Galea et al., 2017; Lymer et al., 2017).

Various cognitive functions may fluctuate during the various phases of the menstrual/estrous cycle, and many studies have examined and highlighted the role of sexual hormones in memory, both in women and female animals (Gasbarri et al., 2008; Konishi et al., 2008; Pompili et al., 2010; Joseph et al., 2012; Hampson and Morley, 2013; Bayer et al., 2014).

On this basis, the principal aim of this study was to analyze the influence that estrogens may have on early memory processing of primary emotional faces. To this purpose, the performance on a recognition memory (RM) task and the reaction time (RT) obtained were compared within subjects for stimulus type (emotional facial expressions) and between subjects for menstrual cycle phase (low and high estrogen levels). P300 responses to emotional faces used as stimuli were analyzed, and electrophysiological and behavioral indices were used to determine group differences in memory processing.

In previous studies in our laboratories, we have analyzed working memory for emotional stimuli consisting of facial expressions using the delayed matching-to-sample task (Gasbarri et al., 2008). We considered a rather large follicular time window in the menstrual cycle, and comprising the lutein phase, in which the effects of progesterone are also present. In this work, in order to analyze the effect of estrogens alone, we restricted the study windows to very limited periods, in particular by including the periovulatory (PO) period, during which the level of estrogens is at its highest, but the level of progesterone is low. Moreover, we used the event-related potential (ERP) method. To our knowledge, there are few studies examining the processing of emotional facial expressions during the menstrual cycle in physiological conditions (Pearson and Lewis, 2005; Derntl et al., 2008a,b; Gasbarri et al., 2008), in particular considering memory processing, and all have differing results.

Here, we intend to better analyze the role of estrogens in physiological conditions on the interplay between cognition and emotion, that is on the interaction between short-term memory and socially relevant stimuli.

The influence of emotion on memory has been abundantly demonstrated using various types of materials, including emotional stories (Gasbarri et al., 2005; Nielsen et al., 2013), pictures (Pompili et al., 2016), and words (Arnone et al., 2011). However, images of emotional faces differ from other arousing pictures or words because of their affective value (Calvo and Nummenmaa, 2016). In fact, primary emotional faces are recognized as having an adaptive role, so they influence the attentive, perceptive, and mnemonic processes because they provide social feedback to themselves; a face can help us to get important information needed during social interactions (Crivelli and Fridlund, 2018), such as race, sex, identity, and the ability to distinguish friends from foes. Consequently, all faces, even those defined as “inexpressive” or “neutral,” can have emotional salience (Palermo and Rhodes, 2007). The perception of a facial emotion involves both visual processing and the recognition of its emotional meaning (Brenner et al., 2014). Understanding a facial expression, therefore, involves the combination of the cognitive processing of the visual stimulus with a memory related to a specific emotion, and it is a skill present from the earliest stages of life; that is, it is hereditary (Grossmann and Johnson, 2007).

Based on our previous study (Pompili et al., 2016), we predicted that healthy young women in the menstrual phase with higher estrogen levels would exhibit a better memory performance, compared to women with low estrogen levels. Moreover, we also hypothesized that hormonal fluctuations could modulate both the electrophysiological and the behavioral response to the different emotional faces.

## Materials and Methods

### Subjects

Women aged 19–25 (mean age 22.7 ± 2.2), all University of L’Aquila students, were recruited to participate in this study. They were initially submitted to a screening interview to check for any health problems, and ensure the regularity of their menstrual cycle. After the screening, 78 subjects were selected, with a menstrual cycle length from 26 to 30 days. Exclusion criteria included the use of any form of hormonal contraceptives in the 4 months before the test, psychiatric illnesses (including premenstrual syndrome), neurological disorders, and chronic illnesses treated with hormonal therapy. Women who were pregnant or lactating in the 12 months before the test were excluded from the experiments. All subjects were right-handed and had normal or corrected-to-normal vision. During the screening interview, data regarding participants’ last menstrual period were collected, and the women were divided into two groups. The early follicular (EF) group included all subjects who were involved in the test between the 1st and the 3rd day of their menstrual cycle, while the PO group included all subjects who participated in the test between the 12th and the 16th day of their menstrual cycle. The ovarian function and the menstrual cycle phase were verified with salivary measures. A salivary sample was collected from every subject 5 min before the test, and later analyzed to evaluate its estradiol and progesterone levels. All the subjects who showed low levels of both estradiol (<2 pg/ml) and progesterone (<75 pg/ml) were included in the EF group, while subjects with high levels of estradiol (>6 pg/ml) but low levels of progesterone (<100 pg/ml) were included in the PO group. Eight subjects were excluded because their tests showed that they were out of the expected hormonal range at the time of testing (three in the EF group and five in the PO group). Four subjects were excluded due to problems found in the EEG acquisition. Therefore, the final analysis included 33 subjects in the EF group and 33 subjects in the PO group. This study was conducted in accordance with the Declaration of Helsinki, and was approved by the ethical Committee of the University of L’Aquila. All the procedures were carried out with the adequate understanding of the subjects, who read and signed an informed consent form before participating in this research project.

All the data were grouped and analyzed anonymously.

### Hormone Salivary Levels

To confirm the phase of the menstrual cycle, participants’ hormonal levels were measured using a competitive enzymatic immunoassay kit (Salimetrics, State College, PA, USA). Salivary levels of estradiol and progesterone are believed to accurately represent the biologically active fraction in general circulation, and this represents a suitable non-invasive method. See Gasbarri et al. (2008) for the test principle, saliva sample assays, and data analysis.

### Stimuli

We utilized stimulus materials selected from the Picture of Facial Affect (POFA) in Ekman and Friesen (1976), a set of human facial expressions widely used in neuropsychological research. Every model in POFA displayed one of the six universal emotions (happiness, anger, disgust, sadness, surprise, fear) or one neutral expression.

**Figure 1:**
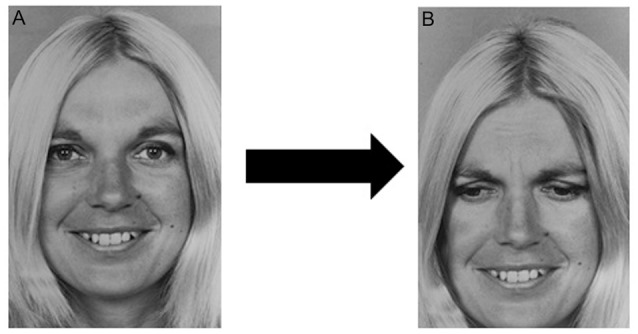
Chimerical image. **(A)** Original stimulus: one of the selected Picture of Facial Affect (POFA) faces showing happiness. **(B)** One of the chimerical images obtained by mixing the upper half of **(A)** with the lower half of the same female model showing a different emotion, in this example case sadness.

We selected 89 stimulus images from the POFA and then used a photo editing software to obtain other 89 chimerical images. Each chimerical image was prepared by mixing the lower or upper part of the stimulus image (e.g., happiness), with the upper or lower part of another stimulus of the same female or male model (e.g., sadness), as shown in Figure 1. The chimerical images were used in the target image, containing both the stimulus just seen and the relative chimerical picture.

The figures used in the experiment procedure were 84, while the remaining five images, not considered in the statistical analysis, were used as training at the beginning of the procedure recording. The 84 experimental pictures were 12 examples for each emotion, six represented by six different male models and six represented by six different female models.

### Procedure

Participants viewed the selected faces while seated in a comfortable chair in a sound-attenuated, dimly lit room. After electrode attachment and laboratory adaptation, they were told that a series of slides representing human faces would be presented and that they should observe each picture the entire time it appeared on the screen, without moving their eyes. All experiments were conducted at 10:00 AM. Every subject was submitted to a RM behavioral task. Participants observed a total of 178 images, the first 10 of which for training purposes, 89 stimuli, and 89 targets, presented with the use of the Presentation software (ver. 0.51).

Every stimulus image, represented by one of the faces selected from POFA, was followed by a target image consisting of the same image and its relative chimerical image.

**Figure 2:**
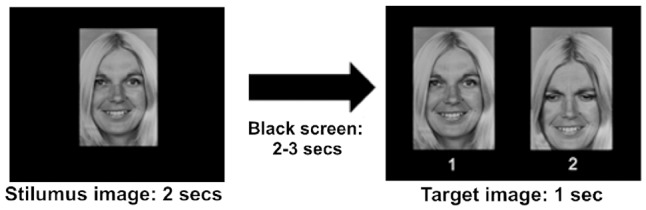
Timeline showing the course of the experiment. The stimulus image was presented for 2 s, followed by a black screen for 2 or 3 s, and finally by a target image. 1: stimulus image; 2: chimerical image. The correct answer in the recognition memory (RM) behavioral task required to choose the original stimulus (1) of the target image.

All the 178 pictures were presented in random order on a Philips 200P4SS 20.1^′′^, 0.25 mm (Dot pitch), with a horizontal rate of 94 Khz and a vertical rate of 85 Hz, and a resolution of 1,280 × 1,024 pixels, positioned 1 m in front of the subject. In the RM behavioral task, the stimulus image was shown on the monitor for 2 s, followed by a black screen of a random variable duration between 2 and 3 s to avoid a habituation effect and then followed by a target image for 1 s (Figure 2). During the projection of the target stimulus, the subject was instructed to give the correct answer by pushing one of the buttons (number 1 or number 2) located on the chair armrests to avoid muscular movements that could generate artifacts in the EEG recording. As mentioned above, the target stimulus contained the emotional face just seen and a chimerical image: the correct answer required to recognize the stimulus just seen (button 1), while pressing the button 2 (chimerical image) was considered an error (Figure 2). Each experimental session had a duration of 20 min, including the training session.

During the EEG recording, the exact moment of presentation of an image, the difference between the stimulus image and target image, the emotion shown, and the answer given by the subject were selected by means of specific triggers in the Presentation software. Values between 11 and 24 were utilized to define emotion and gender differences on the stimulus images; values between 25 and 38 defined the same variables on the target images. Triggers 1 and 2 were utilized to indicate the answers given by the subjects; these triggers allowed the correct segmentation of the EEG recording and its subsequent analysis. The PC utilized for the image presentation (Intel Pentium IV—1.80 Ghz—1 GB RAM) was connected to the PC utilized for the EEG recording (using the same specifications) with a Centronics I/O parallel port.

### EEG Recording

EEG signals were recorded at 30 scalp sites (Fp1, Fp2, F7, F3, Fz, F4, F8, Fc5, Fc1, Fc2, Fc6, T7, C3, Cz, C4, T8, TP9, CP5, CP1, CP2, CP6, TP10, P7, P3, Pz, P4, P8, O1, O2, and Oz) according to the international 10/20 system, and employing ActiCap Control Software (Brain Products GmbH). Channels 31 and 32 were used to assess eye movements, and horizontal and vertical electrooculogram (EOG), respectively. The EOG activity was used to reduce artifacts. The EEG equipment (BRAINAMP—Brain Products GmbH) included the Vision Recorder software, which measures with high-precision GND (ground) and REF (reference) electrode impedance. The impedance of each electrode was checked at ≤1 kΩ. The EEG from each electrode site was digitalized at 250 Hz.

### EEG Analysis

BrainAmp Vision Analyser Software—ver. 1.05.0001—Brain Products GmbH was employed in the EEG analyses. The averaged epochs were 100 ms before and 1,000 ms after the original stimulus onset. Artifacts (deviation in eye position, blinks or amplifier blocking) were removed prior to signal averaging; we used a difference criterion with a minimal-maximal allowed absolute difference of two values in the segment of 200.00 μV. P300 latency was defined as the maximum positivity between 250 ms and 400 ms. We applied a pre-stimulus baseline correction selecting a pre-stimulus windows from −200 to 0 ms relative to stimulus onset. Base-to-peak amplitude (2,000 ms pre-stimulus baseline) and peak latency value of P300 components of the averaged ERPs were subjected to statistical evaluation. To reduce high-frequency noise, the averaged visual evoked potentials were filtered at 0.01 Hz (48 dB/octave) and 35 Hz (48 dB/octave). An Ocular Correction was applied to remove eye blinks, and EOG H and EOG V activity were employed as a sample to filter residual eye-movement artifacts. A segmentation was applied to the EEG recordings based on the specific triggers configured using the Presentation software, obtaining 14 sets of segments, each of which referred to a distinct emotional face. The analysis of P300 components for the stimulus images was determined for parietal electrodes, P3/4, P7/8, and Pz sites, where amplitude and latency were largely evident (Katayama and Polich, 1999).

### Statistical Analysis

Data were analyzed using SPSS, version n. 20. We verified the distribution of both hormonal levels and parameters of the P300, amplitude and latency, before choosing the statistical tests to be applied: all data were normally distributed. Hormonal levels in the different phases were compared using Student’s *t*-test. P300 parameter values amplitude and latency were examined using repeated measures of ANOVA with two electrode site (fronto-parietal and parietal), seven stimuli (happiness, anger, disgust, sadness, surprise, fear and neutral) and two lateralizations (left and right) as within-subjects factors, and cycle phase (PO and EF) as the between-subjects factor. We used one-way ANOVAs to analyze the data related to a specific phase. The *post hoc* planned comparison was performed using Tukey’s Honestly Significant Difference (HSD) test. We also investigated the relationship between hormone levels and RM errors, by means of correlation analyses.

Results are presented as means ± standard error of the mean (SEM), or as means ± standard deviation (SD). The level of significance was set at 0.05.

## Results

### Hormonal Levels

**Figure 3:**
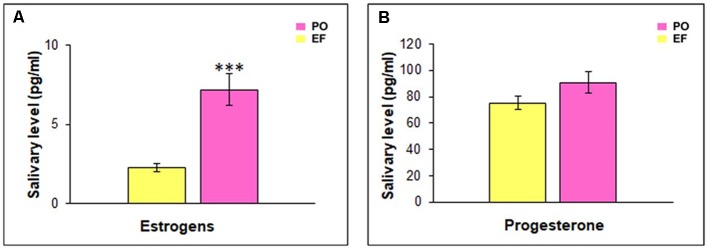
Hormonal salivary levels. **(A)** Estrogen levels were significantly higher in the PO than in the EF group, ***p < 0.001. **(B)** Progesterone levels did not show any significant difference between the two groups.

The EF phase showed low levels of both progesterone (70.19 ± 4.82 pg/ml) and estrogens (1.67 ± 0.3 pg/ml). During the PO phase, progesterone levels remained low (90.75 ± 8.59 pg/ml), while estrogen levels rose at a value of 7.18 pg/ml (SEM = 0.85). A statistical comparison between the hormonal levels characterizing the two groups showed a significant difference related to estrogens (*t*_(64)_ = −5.427; *p* < 0.001), while there was no significant difference in progesterone levels (*t*_(64)_ = −1.546; *p* = 0.127, n.s.; Figure 3).

### Analysis of Event-Related Potentials: P300 Parameters

**Figure 4:**
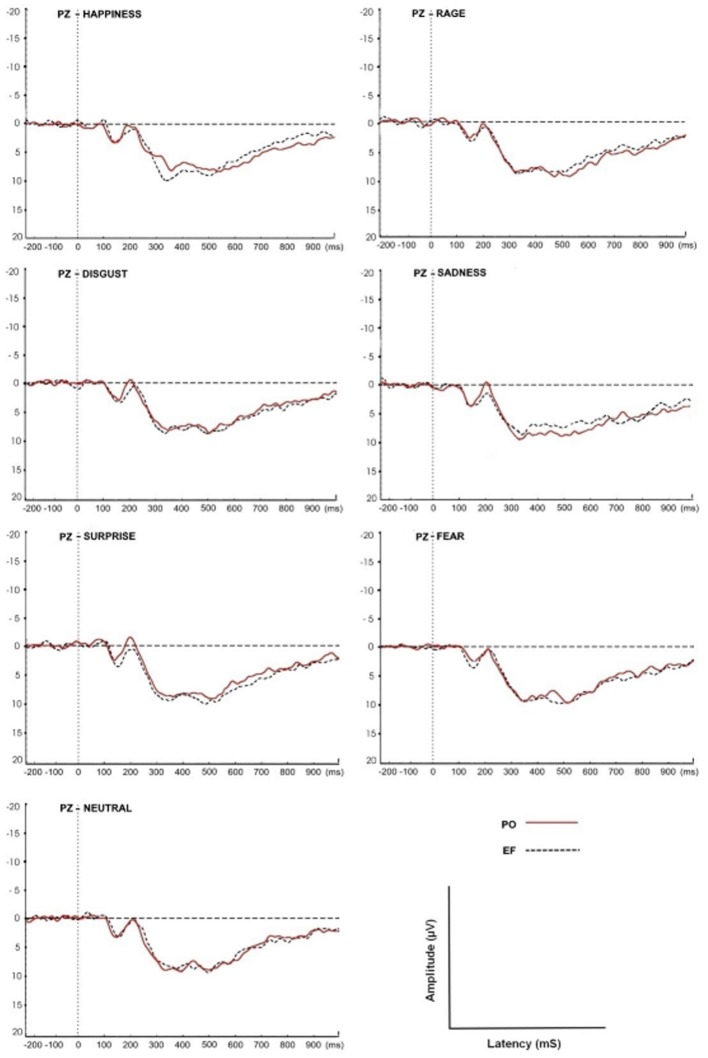
Grand-average event-related potential (ERP) waveforms of all subjects at the Pz site in response to the seven facial expressions. The P300 amplitudes and latencies were determined for both EF and PO groups.

For both the EF and PO phases, the grand-average ERPs of all subjects at the Pz site in response to the seven emotional facial expressions are shown in Figure 4.

### P300 Amplitude

The statistical analysis of the P300 amplitude showed a cycle phase effect: women in the PO phase displayed a larger amplitude compared with those in the EF phase (*F*_(1,68)_ = 5.521; *p* < 0.02).

**Figure 5:**
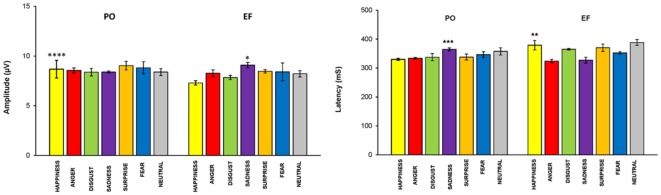
P300 parameters: EF phase vs. PO phase. Means (±SEM) of P300 amplitude and latencies in response to the seven emotional facial expressions during the EF and PO phases. Participants in the PO phase, compared to those in the EF phase, showed a significantly higher amplitude and a significantly shorter latency for the emotion of happiness, and the opposite for sadness. There were no significant differences for the other emotional facial expressions. *p < 0.044; **p < 0.02; ***p < 0.01; ****p < 0.001.

The main effect of the emotional stimuli was also identified. In fact, in the interaction between the cycle phase and emotional facial expressions, women in PO had a higher amplitude, compared to EF, for the stimuli of happiness (*F*_(1,8)_ = 33.087; *p* < 0.001) and a lower response for sadness (*F*_(1,8)_ = 5.662; *p* < 0.044). There were no significant differences for the other emotional facial expressions. The amplitude values for each emotion and group are shown in Figure 5.

### P300 Latency

A cycle phase effect was also found for P300 latency. In fact, women in the PO phase displayed shorter latencies compared with the EF subjects (*F*_(1,68)_ = 4.230; *p* < 0.04). The main effect of the emotional stimuli was demonstrated: latency was significantly lower during PO for happiness (*F*_(1,8)_ = 8.419 *p* < 0.02), but significantly higher for sadness (*F*_(1,8)_ = 11.107, *p* < 0.01; Figure 5). No significant differences in latency were found for the other emotional facial expressions.

### Recognition Memory Task

**Figure 6:**
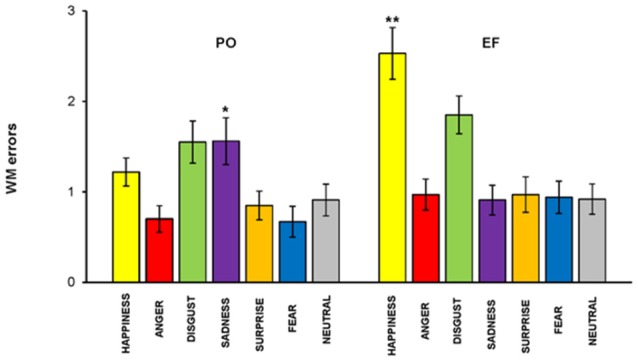
RM task. Subjects in the PO phase made a significantly lower number of RM errors for happiness but a significantly higher number for sadness. There were no significant differences for the other emotions. *p < 0.03; **p < 0.001.

The main effect of the cycle phase was shown for the total number of errors. An overall ANOVA performed on the total number of RM errors, revealed a memory by phase interaction: subjects in the PO phase made a significantly lower number of RM errors (1.05 ± 0.72) compared to the EF phase (1.31 ± 0.86; *F*_(1,460)_ = 6.228, *p* < 0.01). RM errors were significantly lower for happiness (*F*_(1,64)_ = 16.191; *p* < 0.001) and significantly higher for sadness (*F*_(1,64)_ = 4.824; *p* < 0.03) in PO phase compared with the EF phase. No significant differences were found for the other emotional facial expressions (Figure 6).

We performed linear correlations between hormonal levels as measured in saliva and RM errors, to further test these results. We found that estrogen levels were positively correlated with RM errors for sadness, *r*_(33)_ = 0.510, *p* < 0.002, while they were inversely correlated with RM errors for happiness, *r*_(33)_ = −0.619, *p* < 0.000. No significant relationship was found between estrogen levels and the other five emotions, nor between progesterone levels and the other seven emotions.

### Reaction Time

**Figure 7:**
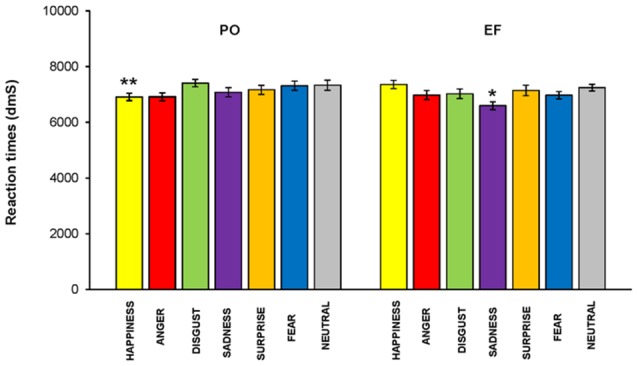
Reaction times (RTs). RTs were significantly lower in the PO phase than those recorded in the EF phase for the emotion of happiness, while they were significantly higher for sadness. There were no significant differences for the other emotions.*p < 0.03; **p < 0.02.

No main effect of the cycle phase on RT was revealed (*F*_(1,460)_ = 1.910; *p* = 0.168, n.s.); however, RTs were significantly lower in PO than in the EF phase for the emotion of happiness (*F*_(1,64)_ = 5.140; *p* < 0.02), while they were significantly higher for sadness (*F*_(1,64)_ = 5.031; *p* < 0.03). There were no significant differences for the other emotions (Figure 7).

## Discussion

In the present study, we investigated the effect of estrogens on the short-term memory processing for emotional facial expressions in healthy young women during different times of their menstrual cycle: the EF and PO phases. To meet this aim, we employed an RM task, during which ERPs in response to emotional facial expressions were recorded and P300 components, amplitudes, and latencies were considered. Data from ERPs, memory performance, and RT were used to draw conclusions about the role of estrogens in processing emotional facial expressions and subsequent memory encoding.

The enzyme immunoassays performed on saliva samples provide evidence of a significantly higher estradiol concentration in the PO phase (12th–16th day) compared to the EF phase (1st–4th day). An increase in estradiol levels was therefore highlighted, as expected, right at the ovulation peak, while progesterone levels remained low in both phases. Therefore, the hormonal analyses allow us to hypothesize that the modulations observed during our experiments, related both to ERPs and to behavioral performances, could be linked to fluctuations in hormonal estrogen levels, rather than progesterone ones.

Our main findings, showing an interaction between estrogen levels and electrophysiological and behavioral performances, were that subjects in the PO phase, compared to those in the EF phase, displayed: (i) a significantly greater amplitude of P300, and a significantly shorter latency for the emotional faces expressing happiness, but the opposite for sadness. No significant differences were exhibited for the other facial expressions; (ii) in the RM task, there was a better performance, with fewer statistically significant errors in the processing of happiness, but the opposite for sadness; and (iii) finally, according to the previous results, the RT phase was significantly faster for happiness, and the opposite for sadness.

Overall, these findings indicated that subjects had a better performance in the PO phase, compared to the EF phase, while viewing facial expressions of happiness, showing a significant lower reactivity to sadness. After indicating that a higher P300 amplitude and a lower P300 latency in response to the original stimulus were associated with the better recognition of the target images, the results showed that participants paid greater attention to the specific stimuli of happiness. The choice to evaluate the P300 component was based on its well-known role as an index of the cognitive abilities that a subject invests in the execution of a task. From a theoretical point of view, the increase in amplitude, usually inversely linked to latency, shows a greater investment of attentive resources in a task (Polich and Kok, 1995). The P300 was thought, essentially, to reflect memory ability, in particular, greater allocation of cognitive resources to more demanding memory operations (Polich, 2007). When a stimulus was presented to a subject, during electroencephalographic procedure, a positive potential was elicited that increases in amplitude from the frontal to the parietal electrodes (Johnson, 1993). For this reason, we analyzed the parietal electrode sites, where the activity is more pronounced (Katayama and Polich, 1999).

The existing literature has evidenced a relationship between the P300 component of the ERPs and emotional arousal (for a comprehensive review see Olofsson et al., 2008; Güntekin and Başar, 2014), and research conducted in both our laboratory and those of others reported that emotionally arousing stimuli elicited greater P300 responses than neutral stimuli at encoding (Dolcos and Cabeza, 2002; Keil et al., 2002; Gasbarri et al., 2006, 2007; Pompili et al., 2016; Osugi and Ohira, 2018). Emotional stimuli might automatically be processed as relevant because of their intrinsic motivational significance; since in our task six of the seven stimuli were of the emotional type, it is possible to believe that joy is considered a more relevant emotional stimulus from a motivational point of view. In support of this, the fact that sadness, which we could consider to be the opposite of joy, obtained the lowest responses is evidenced.

Some studies investigated, without the use of ERPs, the perception and recognition of specific emotional expressions during different phases of the menstrual cycle (Conway et al., 2007; Guapo et al., 2009; Kamboj et al., 2015). In their works focused on emotional recognition, Derntl et al. (2008a,b) found greater accuracy in the follicular phase than in the luteinic phase, with a negative correlation between progesterone levels and the accuracy of emotional recognition. There is experimental evidence on the role of estrogens in the recognition of emotions. Pearson and Lewis (2005), in their study, in which the accuracy of the recognition of emotional expressions was examined, found a specific association between the phases of the menstrual cycle and the recognition of fear. Women were more accurate in recognition during the preovulatory phase (i.e., around ovulation) when estrogen levels are high, rather than during menstruation.

Zhang et al. (2013), in their study on the time course of facial expression recognition during the menstrual cycle, found that hormonal fluctuations during menstrual cycle modulated only the late positive potential (LPP2, from 750 to 1,000 ms after the stimulus presentation) in response to emotional faces, with a larger amplitude during the periovulation phase, compared to the premenstrual phase. They also found a positive correlation between the LPP2 amplitude and facial expression recognition accuracy during the periovulation phase, but the effect was independent of any specific facial expression. However, the authors did not obtain direct measures of hormone levels.

The principal findings of our study was that, in young women, the physiological fluctuations of estrogens during the menstrual cycle modulate the sensitivity to specific social stimuli of happiness during PO, affecting the processing of memory recognition.

In conclusion, the evaluation of our results, in light of the emotional value of the stimuli, suggests that physiological fluctuations of estrogens during the menstrual cycle should influence one’s preference for the facial expressions of happiness during PO, while sadness was not considered to be a salient stimulus. Considering that in PO the risk of conception is high, estrogens could increase the sensitivity of women to relevant stimuli, from an evolutionary point of view, to help mating behavior; happiness could, therefore, be considered to be a self-relevant stimulus during the ovulatory phase, as opposed to sadness valence, as human facial expressions can regulate approaching and avoiding behaviors with others (Seidel et al., 2010).

The aim of this study was to clarify the role of female sex hormones in cognitive activities, in order to develop new therapeutic strategies to promote successful ageing. Our results, however, should be considered within the limits of the research design, in which we have considered only healthy young women. In fact, since there is an interaction between serotonin, which has an important role in modulating mood, and estrogens, we cannot completely exclude that the preference for faces expressing happiness, unlike sadness, may be due to a greater propensity in the PO phase towards positive stimuli rather than negative, beyond the evolutionary aspect. Estrogens act positively on serotonin transmission in many ways, including the modulation of synthesis and degradation (Smith et al., 2004; Hiroi et al., 2006), the inhibition of reuptake from synapses, the regulation of the density of post-synaptic 5-HT2A ERs (Moses-Kolko et al., 2004), and the sensitivity of the presynaptic 5-HT1A auto receptor (Henderson and Bethea, 2008). The role of estrogens in mood and emotional states in women is strongly suggested by their potentiation of serotoninergic activities in the limbic area (Wharton et al., 2012).

We are now planning studies involving subjects with emotional disorders linked to the menstrual cycle. These kinds of studies can help us to better differentiate between the psychological and physiological aspects of estrogen influence.

## Data Availability Statement

The datasets generated for this study will not be made publicly available because all necessary data are included in the work.

## Ethics Statement

The studies involving human participants were reviewed and approved by Internal Review Board of University of L’Aquila. The patients/participants provided their written informed consent to participate in this study.

## Author Contributions

AG and AP conceptualized and designed the study. MD’A wrote a section of the manuscript and acquired the data. BA, CI, FP and PI acquired data and contributed to data analysis. SC organized the database and contributed to technical support. AP wrote the first draft of the manuscript. All authors contributed to manuscript revision, read and approved the submitted version.

## Conflict of Interest

The authors declare that the research was conducted in the absence of any commercial or financial relationships that could be construed as a potential conflict of interest.
